# Supplementary material for: Wheat-Bran-Based Artificial Diet for Mass Culturing of the Fall Armyworm, Spodoptera frugiperda Smith (Lepidoptera: Noctuidae)
Source: Insects. 2022 Dec 19;13(12):1177. doi: 10.3390/insects13121177 (PMC9788468; doi:10.3390/insects13121177)
Supplement: Supplementary file 1 [file insects-13-01177-s001.zip › insects-2079273-supplementary.pdf]

Table S1. Purchase price of each material in diet

Material	Costs per kg/500 mL (RMB)
Wheat bran	2.40
Soybean powder	16.00
Maize powder	8.00
Yeast powder	70.00
Agar	110.00
Casein	90.00
Sorbic acid	50.00
Ascorbic acid	27.00
Vitamins	4540.00
Formaldehyde	30.00
Glacial acetic acid	6.00

Exchange rate at time of writing: RMB 1 = USD 0.1380.

Table S2. Purchase price of each vitamin in diet.

Material	Costs per g (RMB)
Aneurine hydrochloride	8.00
Riboflavin	1.40
Nicotinic acid	4.00
D-pantothenic acid hemicalcium salt	3.20
Pyridoxine hydrochloride	1.60
Cyanocobalamin	1.60
Folic acid	4.00
Nicotinamide	180.00

Exchange rate at time of writing: RMB 1 = USD 0.1380.
